# A propensity-score matched analysis of ventral-TAPP vs. laparoscopic IPOM for small and mid-sized ventral hernias. Comparison of perioperative data, surgical outcome and cost-effectiveness

**DOI:** 10.1007/s10029-022-02586-x

**Published:** 2022-03-23

**Authors:** I.-F. Megas, C. Benzing, A. Winter, J. Raakow, S. Chopra, J. Pratschke, P. Fikatas

**Affiliations:** Department of Surgery, Campus Charité Mitte and Campus Virchow-Klinikum, Charité, Universitätsmedizin Berlin, Corporate Member of Freie Universität Berlin, Humboldt-Universität Zu Berlin, and Berlin Institute of Health, Augustenburger Platz 1, 13353 Berlin, Germany

**Keywords:** Laparoscopic ventral hernia repair, Preperitoneal, Ventral-TAPP, IPOM, Propensity-score matching

## Abstract

**Purpose:**

Laparoscopic techniques have been used and refined in hernia surgery for several years. The aim of this study was to compare an established method such as laparoscopic intra-peritoneal onlay mesh repair (lap. IPOM) with ventral Transabdominal Preperitoneal Patch Plasty (ventral-TAPP) in abdominal wall hernia repair.

**Methods:**

Patient-related data of 180 laparoscopic ventral hernia repairs between June 2014 and August 2020 were extracted from our prospectively maintained database. Of these patients, 34 underwent ventral-TAPP and 146 lap. IPOM. After excluding hernias with a defect size > 5 cm and obtaining balanced groups with propensity-score matching, a comparative analysis was performed in terms perioperative data, surgical outcomes and cost-effectiveness.

**Results:**

Propensity-score matching suggested 27 patients in each of the two cohorts. The statistical evaluation showed that intake of opiates was significantly higher in the lap. IPOM group compared to ventral-TAPP patients (*p* = 0.001). The Visual Analogue Scale (VAS) score after lap. IPOM repair was significantly higher at movement (*p* = 0.008) and at rest (*p* = 0.023). Also, maximum subjective pain during hospital stay was significantly higher in the lap. IPOM group compared to ventral-TAPP patients (*p* = 0.004). No hernia recurrence was detected in either group. The material costs of ventral-TAPP procedure (34.37 ± 0.47 €) were significantly lower than those of the lap. IPOM group (742.57 ± 128.44 € *p* = 0.001). The mean operation time was 65.19 ± 26.43 min in the lap. IPOM group and 58.65 ± 18.43 min in the ventral-TAPP cohort. Additionally, the length of hospital stay in the lap. IPOM cohort was significantly longer (*p* = 0.043).

**Conclusion:**

Ventral-TAPP procedures represent an alternative technique to lap. IPOM repair to reduce the risk of complications related to intra-peritoneal position of mesh and fixating devices. In addition, our study showed that postoperative pain level, material costs and hospital stay of the ventral-TAPP cohort are significantly lower compared to lap. IPOM patients.

**Supplementary Information:**

The online version contains supplementary material available at 10.1007/s10029-022-02586-x.

## Introduction

Numerous methods for repairing ventral hernias have been reported in the literature. Among the laparoscopic methods, laparoscopic intra-peritoneal onlay mesh repair (lap. IPOM) has been established as a simple and safe method in recent years [1–3]. Currently, the lap. IPOM and open sublay operations are the most commonly used methods [4, 5]. Nevertheless, the lap. IPOM also has been under discussion regarding abdominal adhesions and associated postoperative pain [6, 7]. Unfortunately, postoperative pain remains a continuous clinical problem in many patients, so that the possibility of laparoscopic hernia repair with pre-peritoneal mesh placement is regularly discussed as a potential alternative [8, 9]. This discussion is still ongoing, as the repair of ventral hernias are associated with several possible complications. Of note, small bowel obstruction because of adhesions, mesh infection, erosion, and enterocutaneous fistula are the most relevant complications described, which are presumably due to interaction of the mesh with the visceral organs [10].

An important reference regarding hernia repairs is the International Endohernia Society (IEHS) guidelines [11]. In their current version, both pre-peritoneal and intra-peritoneal repairs are described as adequate methods for the treatment of small to medium-sized ventral and incisional hernias (EHS classification W1 and W2). In these guidelines, the question was raised whether laparoscopic pre-peritoneal ventral and incisional hernia repair is possible [11–13].

The general opinion seems to be that TAPP for ventral hernia repair, while technically demanding and requiring elevated expertise on the part of the surgeon, also seems to have some advantages in terms of cost-effectiveness and location of the mesh [11]. However, current published data regarding these aspects are sparse. Surprisingly, only two comparative studies regarding ventral-TAPP repair and IPOM for abdominal hernias have been published, one of which was in robotic surgery [14, 15].

To the best of our knowledge, this is the first European study comparing lap. IPOM and ventral-TAPP in abdominal hernia repair. This study seeks to evaluate whether there are advantages in pre-peritoneal mesh insertion in ventral-TAPP compared to established lap. IPOM technique.

## Methods

The patient-specific data of laparoscopic hernia repairs at Department of Surgery, Campus Charité Mitte and Campus Virchow-Klinikum, Charité—Universitätsmedizin Berlin, between June 2014 and August 2020 were obtained from our prospectively maintained database. Ethical approval No EA1/067/20 was waived by the Ethics Committee of Charité—Universitätsmedizin in view of the retrospective nature of the study. All authors certify that the study was performed in accordance with the ethical standards as laid down in the 1964 Declaration of Helsinki.

**Fig. 1:**
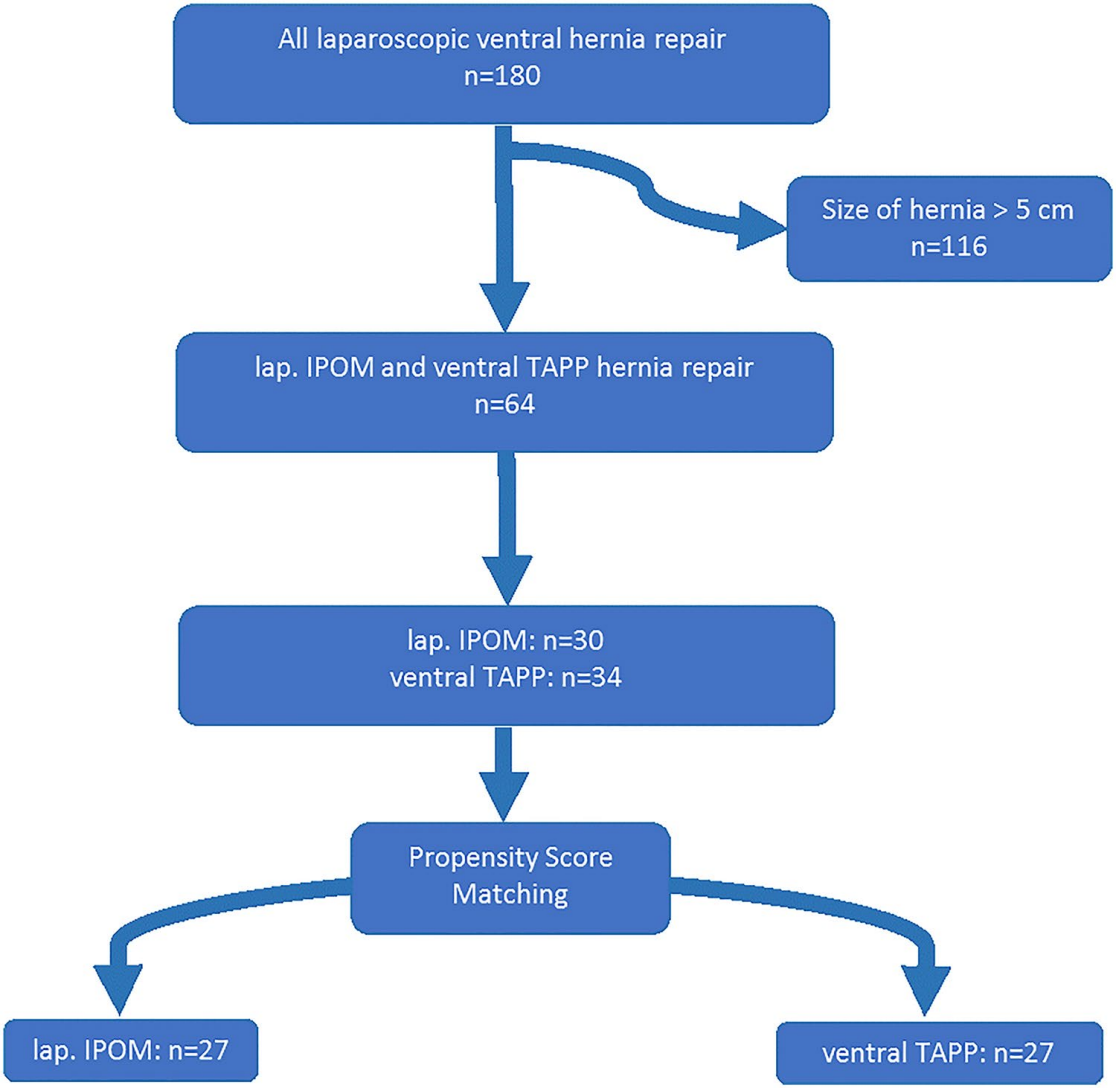
Flow chart of patient selection

From the beginning, the explicit search in our database focused on patients who underwent lap. IPOM or ventral-TAPP repair. More precisely, the other exclusion criteria for this study were: patients who underwent (a) laparoscopically assisted hernia repair with primary closure by suture, (b) hernia repair with a hybrid technique, (c) retro-rectal/retro-muscular mesh repair, or (d) component separation or cases in which lap. IPOM or ventral-TAPP was performed simultaneously with other surgical procedures. The flow chart of patient selection is shown in Fig. 1.

This initial search of our prospectively maintained database revealed 180 cases of hernias repaired by lap. IPOM or ventral-TAPP from 2014 to 2020. Of these patients, 34 underwent ventral-TAPP and 146 underwent lap. IPOM. Since the pre-peritoneal mesh placement is limited by the distribution of peritoneal fat at the lower abdomen and the midline, ventral-TAPP repair cannot be applied in larger hernias. Therefore, we excluded from our analysis hernias with a defect size larger than 5 cm and 30 cases of lap. IPOM repair were left. Statistical analysis was initiated, after balanced groups of 27 patients in each cohort were obtained with propensity-score matching,

Preoperatively known parameters such as demographics (age, gender), comorbidities, body mass index (BMI) and the American Society Anesthesiologists (ASA), the aetiology of the hernia (primary ventral/incisional) and the location, procedure setting (elective/emergent), presence of incarceration were examined. Furthermore, whether closure of the defect was achieved and the type of mesh were the nominal categorical intra-operative data. Continuous numerical intra-operative variables included the dimensions of the hernia defect and the mesh itself and the operating time in minutes. The size of the hernia defects was measured according to the standards of the European Hernia Society [16].

The perioperative parameters investigated in this study were: the occurrence of intra- and postoperative complications such as wound healing disorders or ileus, the length of hospital stay in days and the additional intake of opiates due to severe pain. Our nursing staff and the attending physicians documented daily the postoperative pain at rest and at movement, using a 0–10 Visual Analogue Scale (VAS) scoring system (0: no pain, 10: the worst pain) [17, 18]. The first pain score was obtained on the first day after surgery. The second score included in our evaluation was the maximum postoperative pain. Data on long-term complications were collected in our standardised follow-up routine over 31.96 ± 27.57 months for lap. IPOM and over 14.70 ± 15.76 months for ventral-TAPP and during optional clinical visits after surgery. Those identified in a 3-month interval after surgery were classified according to the Clavien–Dindo System [19].

Finally, the calculation of the costs of the respective surgical method refers to the pure material costs as purchased by Charité—Universitätsmedizin Berlin. For the lap. IPOM method, the costs of the mesh and tacking device were considered. For the ventral-TAPP method, only the mesh was calculated, since mesh was placed between fascia and peritoneum without additional fixation. Other standard-suture material was not considered, as it is the same in both methods.

### Statistical and propensity-score analysis

The descriptive statistics for this study were used to summarize the common, relevant parameters of the patients (demographics, preoperative features like hernia size an comorbidities, operative characteristics and techniques and postoperative outcomes including follow-up features). Depending on the statistical standard for the respective categories, categorical variables (qualitative parameters) were presented as frequency with percentage [*n* (%)] and continuous variables (numerical values) as mean ± SD or median (interquartile range, IQR). The Pearson chi-squared test or Fisher’s exact test were used for the categorical variables. Whereas continuous variables were analysed with the *t* test for independent samples (for normal distributions) and the Mann–Whitney *U* test (for non-normal distributions).

Statistical analysis in this study was carried out using the Statistical Package for Social Sciences software (SPSS Statistics version 25, IBM Corp.), R Studio Desktop version 1.4.1103 and R version 3.3.2 for propensity-score matching. A *p* value of < 0.05 was considered significant.

Propensity-score methods offer certain advantages over more traditional regression methods in observational studies [20]. We used this method to identify balanced, comparable cohorts (lap. IPOM and ventral-TAPP cohort), which is common practice in medical studies [21]. This analysis was performed after excluding the patients with hernia defects > 5 cm. After estimation of the propensity score, we matched participants using a simple 1:1 nearest neighbour matching, without replacement (caliper 0.5). The two groups were compared in terms of peri- and intra-operative variables and postoperative outcomes.

### Surgical techniques

Lap. IPOM and ventral-TAPP repair were performed under general anaesthesia by intubation or laryngeal mask with patients in the supine position. The creation of the pneumoperitoneum was set to an insufflation pressure of 15 mmHg. Access into the abdomen was made using a mini-laparotomy. A total of three trocars were used (two working ports and a single camera port).

### Lap. IPOM procedure

After 360° inspection of the abdominal cavity, all abdominal wall adhesions, if present, were released. After identification, the hernia contents were reduced into the abdominal cavity. Structures surrounding the defect and possibly obstructing mesh placement, such as the peritoneum or the umbilical and falciform ligaments, were dissected. The fascial defect was measured under desufflation. Now, if possible and desired, the primary closure of the hernia defect was performed with interrupted non-absorbable sutures (Ethibond™, UPS 0). The mesh was then deployed and fixed to the posterior fascia with the two provided transfascial non-resorbable sutures and absorbable staples (Securestrap™, Ethicon) using a “double crown” technique.

### Ventral-TAPP procedure

**Fig. 2:**
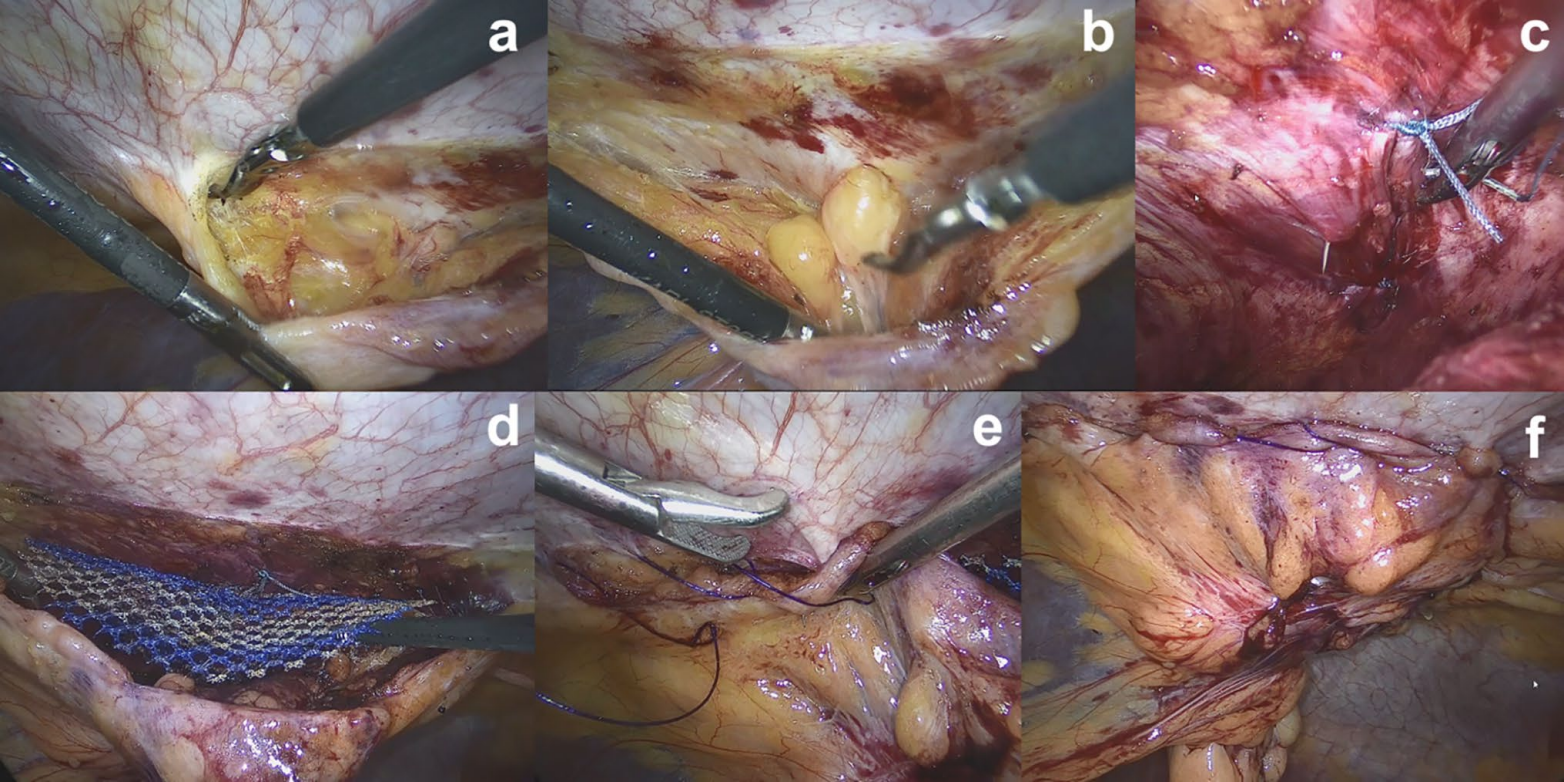
**a** Paramedian incision of the peritoneum, **b** release of hernia sac, **c** closure of the hernia defect with the slipknot, **d** mesh placement, **e** closure of the peritoneum, **f** final complete coverage of the mesh

After establishing a capnoperitoneum of 15 mmHg, an overview of the abdominal cavity was also obtained. Then, as in the lap. IPOM method, adhesions were released. The peritoneum was grasped at least 4 cm from the hernia defect and incised at the left paramedian line, this was done using monopolar scissors and a bipolar grasping forceps (Fig. 2a). The hernia sac with the herniated tissue was released and retracted into the intra-abdominal cavity. (Fig. 2b). To facilitate mesh placement over the defect, a pre-peritoneal area of at least 5 cm in all directions was prepared. Primary closure of the hernia defect was performed using interrupted non-absorbable sutures (Ethibond™, UPS 0). For this surgical step, intra-abdominal pressure was reduced to 8–10 mmHg. The knots were performed using an extracorporeally manufactured slipknot developed by the surgical team and described in patent file WO2016/005118 A1 (Fig. 2c). Next, the mesh was positioned between the posterior fascia and peritoneum. According to the mesh placement at inguinal TAPP repair, no securing sutures of the mesh are necessary (Fig. 2d). If the peritoneum was injured during the preparation, the lesions were also repaired with absorbable sutures. After the mesh was adequately positioned, the peritoneal flap was closed with an absorbable barbed suture (3–0V-Loc™, Medtronic™) (Fig. 2e, f). A video capture of ventral-TAPP procedure is been provided as attachment to the manuscript.

In both techniques, the two working trocars and the camera trocar were removed under visual control and the pneumoperitoneum was released. A fascial closure of trocar sites of > 5 mm diameter was performed with absorbable sutures.

## Results

Due to the size of the hernia defect (> 5 cm), 116 of the initial 180 patients identified in our database as lap. IPOM or ventral-TAPP were excluded. This heterogeneous cohort of patients before propensity-score matching was as follows: 64 patients (mean age 55.36 ± 13.26, 64.1% male). Of these, 46.9% (*n* = 30) underwent lap. IPOM and 53.1% (*n* = 34) underwent ventral-TAPP. The propensity-score matching of the two techniques included patient demographics, as age and BMI, standardisation of comorbidities in form of the ASA score and hernia size. As a result, 27 patients each were assigned to the two now balanced and comparable groups (54 in total—mean age 56.35 ± 12.793, 68.5% male). Both cohorts were considered for our statistical analysis, the matched groups as well as the non-matched groups. Demographics and hernia characteristics data of unmatched and matched cohorts are provided in Table 1. After matching, we examined the overall balance to test the adequacy of our matching. The overall balance test was not significant, confirming that our groups were appropriately distributed.

**Table 1:** Comparison of baseline characteristics of the lap. IPOM and ventral-TAPP groups before and after propensity-score matching

Demographics and hernia characteristics	Unmatched comparisons			Propensity matched comparisons		
	Lap. IPOM (*n* = 30)	Ventral-TAPP (*n* = 34)	*p* value	Lap. IPOM (*n* = 27)	Ventral-TAPP (n = 27)	*p* value
Age (years) mean ± SD	55.83 ± 11.6	54.94 ± 14.70	0.791	55.48 ± 11.72	57.22 ± 13.95	0.622
Sex—male [*n* (%)]	19 (63.3)	22 (64.7)	0.91	18 (66.7)	19 (70.4)	0.769
BMI (kg/m^2^) mean ± SD	32.15 ± 6.66	30.35 ± 5.82	0.252	31.65 ± 6.28	30.84 ± 5.93	0.626
ASA score						
ASA I [*n* (%)]	1 (3.3)	6 (17.6)	0.05	1 (3.7)	2 (7.4)	0.593
ASA II [*n* (%)]	17 (56.7)	22 (64.7)		17(63.0)	19 (70.4)	
ASA III [*n* (%)]	12 (40.0)	6 (17.6)		9 (33.3)	6(22.2)	
Procedure setting						
Elective [*n* (%)]	30 (100)	34 (100)		27 (100)	27 (100)	
Emergency [*n* (%)]	0 (0)	0 (0)		0 (0)	0 (0)	
Hernia aetiology						
Primary ventral [*n* (%)]	19 (63.3)	26 (76.5)	0.251	17 (63.0)	23 (85.2)	0.283
Epigastric	2 (6.7)	1 (2.9)		1 (3.7)	1 (3.7)	
Epigastric and umbilical	2 (6.7)	4 (11.8)		2 (7.4)	4 (14.8)	
Umbilical	14 (46.7)	21 (61.8)		13 (48.1)	18 (66.7)	
Spigelean hernia	1 (3.3)	0 (0)		1 (3.7)	0 (0)	
Incisional [*n* (%)]	11 (36.7)	8 (23.5)		10 (37.0)	4 (14.8)	
Incarceration [*n* (%)]	0 (0)	0 (0)		0 (0)	0 (0)	1

Another way of checking the successful propensity-score matching was to compare the size of the hernia defect. Here, our study showed no significant difference between the two groups investigated (*p* = 0.206), which is also reflected in the EHS classification and thus certified adequate propensity-score matching for our research question.

There was no emergency lap. IPOM or ventral-TAPP in our cohort. All surgeries were electively planned. Surgical time was slightly longer in the lap. IPOM group (65.19 min) than in the ventral-TAPP group (58.65 min), without statistical significance (*p* = 0.3). Primary defect closure was performed more frequently in the ventral-TAPP group than in the lap. IPOM group, according to the surgeon’s preference and the IEHS guidelines in 2019 (7.4% vs. 100%, *p* = 0.001) [12, 13]. Regarding the choice of meshes, coated meshes were used for the lap. IPOM method. 92.6% of lap. IPOM repairs were performed with Parietex™ Optimised Composite (PCOx) Mesh (Medtronic™), 3.7% with PHYSIOMESH™ ETHICON and 3.7% with PROCEED™ Surgical Mesh ETHICON™. In the ventral-TAPP group, Optilene™ MESH, B Braun was used in all cases, which are not coated as there is no contact with the intra-abdominal organs in this method. There was a significant difference (*p* = 0.001) in the size of the mesh used in the two groups 199.33 ± 28.22 cm^2^ versus 87.19 ± 49.07 cm^2^ for lap. IPOM and ventral-TAPP, respectively. This is due to the fact, that the mesh in the ventral-TAPP method was customized and the mesh in the lap. IPOM method was placed in the original size as defined by the manufacturer’s intended use. The matched und unmatched comparison of perioperative details between lap. IPOM and ventral-TAPP groups are provided in Table 2.

**Table 2:** Comparison of operative details between lap. IPOM and ventral-TAPP groups before and after propensity-score matching

Intraoperative variable comparison	Unmatched comparisons			Propensity matched comparisons		
	Lap. IPOM (*n* = 30)	Ventral-TAPP (*n* = 34)	*p* value	Lap. IPOM (*n* = 27)	Ventral-TAPP (*n* = 27)	*p* value
Hernia size (cm^2^) mean ± SD	3.45 ± 1.18	2.747 ± .98	**0.012**	3.35 ± 1.17	2.98 ± .945	0.206
Mesh size (cm^2^) mean ± SD	197.10 ± 27.57	84.74 ± 47.48	**0.001**	199.33 ± 28.22	87.19 ± 49.07	**0.001**
Operating time (min) mean ± SD	65.33 ± 25.39	57.61 ± 18.36	0.169	65.19 ± 26.43	58.65 ± 18.43	0.303
Types of mesh used						
Optilene® Mesh, Braun™ [*n* (%)]	0 (0)	34 (100)		0 (0)	27 (100)	
Parietex™ Composite Mesh, Medtronic™ [*n* (%)]	28 (93.3)	0 (0)		25 (92.6)	0 (0)	
PHYSIOMESH™ ETHICON™ [*n* (%)]	1 (3.3)	0 (0)		1 (3.7)	0 (0)	
PROCEED™ Mesh, ETHICON™ [*n* (%)]	1 (3.3)	0 (0)		1 (3.7)	0 (0)	
Intraoperative complications [*n* (%)]	0 (0)	0 (0)		0 (0)	0 (0)	
Material costs (€) mean ± SD	733.41 ± 124.79	34.34 ± 0.42	**0.001**	742.57 ± 128.44	34.37 ± .47	**0.001**
Diastasis recti [*n* (%)]	1 (3.3)	4 (11.8)	0.179	1 (3.7)	2 (7.4)	0.552
Primary defect closure [*n* (%)]	3 (10.0)	33 (97.1)	**0.001**	2 (7.4)	27 (100)	/
EHS (width)						
w1	24 (80.0)	30 (88.2)	0.365	23 (85.2)	23 (85.2)	1
w2	6 (20)	4 (11.8)		4 (14.8)	4 (14.8)	
w3	0 (0)	0 (0)		0 (0)	0 (0)	

Statistically significant results are marked in bold

Intraoperatively, there were no complications in our study. Postoperatively, there were significant differences between the groups in terms of analgesic medications used. The use of intravenous and oral opiates was significantly higher in the lap. IPOM cohort (*p* = 0.001). Furthermore, all pain scores were significantly higher in the lap. IPOM group (Table 2). More specifically, the mean pain score on 1st postoperative day (POD) at rest and on movement was analysed. According to the 0–10 scale system, VAS score was 2.28 ± 1.275 (rest) and 3.32 ± 1.49 (movement) in the lap. IPOM group and 1.33 ± 1.18 (rest) and 2.26 ± 1.75 (movement) in the ventral-TAPP group. Pain level at this time point showed statistical significance between the two groups (*p* = 0.008 at rest and *p* = 0.023 at movement, respectively). Regarding the maximum pain sensation during the hospital stay, there was also statistical significance between the two groups. The maximum VAS score was significantly higher in the lap. IPOM group compared to ventral-TAPP patients (3.76 ± 1.45 vs. 2.48 ± 1.58, *p* = 0.004).

Within the first three months, only first-degree complications were observed according to the Clavien–Dindo classification. The distribution was as follows: in the lap. IPOM group, three patients (11.1%) had a grade I complication, while in the ventral-TAPP group grade I was observed in two patients (7.4%). In the ventral-TAPP cohort, one patient reported wound healing problems in the follow-up, which had already healed secondarily at the time of the examination. The other case documented according to the Clavien–Dindo classification reported diffuse abdominal pain for which no corelate was found.

The lap. IPOM cohort showed an organised haematoma, an unclear swelling and one case of pain most likely due to the mesh fixation with the tacks. The discrete numerical difference in this category showed no statistical relevance (*p* = 0.639).

In terms of cost-effectiveness, the analysis showed extraordinary difference between the two study groups. The material costs of the ventral-TAPP procedure were 34.37 ± 4 €, significantly lower than those of the lap. IPOM group 742.57 ± 128.44 € (*p* = 0.001). Note, even if not all tackers were used for mesh fixation, as a single use instrument the whole device price has to be considered in cost calculation analysis. Additionally, the length of hospital stay in the lap. IPOM cohort was significantly longer (2.81 ± 0.88 vs. 2.37 ± 0.69, *p* = 0.043). This almost half day shorter hospital stay after ventral-TAPP procedure is an additional parameter of cost-effectiveness, which, however, differs from clinic to clinic and from country to country, depending on health system regulations.

Finally, none of the patients experienced hernia recurrence during the follow-up period (31.96 ± 27.57 for lap. IPOM group and 14.70 ± 15.76 months for ventral-TAPP, respectively) of this study. Outcome results from matched and unmatched analysis is shown in Table 3.

**Table 3:** Postoperative outcomes before and after propensity-score matching

Postoperative outcomes	Unmatched comparisons			Propensity matched comparisons		
	Lap. IPOM (*n* = 30)	Ventral-TAPP (*n* = 34)	*p* value	Lap. IPOM (*n* = 27)	Ventral-TAPP (*n* = 27)	*p* value
VAS score mean ± SD						
Pain at rest—1st POD	2.25 ± 1.24	1.31 ± 1.12	**0.003**	2.28 ± 1.275	1.33 ± 1.18	**0.008**
Pain of movement—1st POD	3.29 ± 1.44	2.25 ± 1.67	**0.013**	3.32 ± 1.49	2.26 ± 1.75	**0.023**
VAS max. hospital stay	3.68 ± 1.42	2.47 ± 1.50	**0.002**	3.76 ± 1.45	2.48 ± 1.58	**0.004**
Opiate intake [*n* (%)]	16 (53.3%)	4 (11.8%)	**0.001**	14 (51.9%)	3 (11.1%)	**0.001**
Early complications [*n* (%)]	0 (0)	0 (0)		0 (0)	0 (0)	
Late complications						
Surgical-site events [n (%)]	1 (3.3)	1 (2.9)	0.559	0 (0)	0 (0)	1
Hematoma [*n* (%)]	1 (3.3)	0 (0)		1 (3.7)	1 (3.7)	
Hospital stay (days) mean ± SD	2.87 ± 0.860	2.32 ± 0.638	**0.005**	2.81 ± 0.88	2.37 ± 0.69	**0.043**
Follow-up (months)	31.63 ± 26.57	14.53 ± 15.02		31.96 ± 27.57	14.70 ± 15.76	
Recurrence	0 (0)	0 (0)		0 (0)	0 (0)	
Clavien–Dindo						
Grade I	4 (13.3%)	3 (8.8%)		3 (11.1%)	2 (7.4%)	0.639
Grade II, III, IV	0 (0)	0 (0)	0.564	0 (0)	0 (0)	

Statistically significant results are marked in bold

## Discussion

Ventral hernias are a common condition and have been treated for years as a standard and highly frequented procedure. Lap. IPOM and open sublay repair have become established over the years and currently are the most frequently used for the treatment of small to medium-sized primary and incisional abdominal wall hernias [4].

The existing literature shows that lap. IPOM repair is associated with fewer infections and wound healing complications compared to open mesh repair [2, 4, 22]. Therefore, refinement and optimisation of laparoscopic alternatives is an area of interest. However, the lap. IPOM technique does not seem to have any advantage in terms of postoperative pain compared to the open procedures [23]. Furthermore, there is still the problem that the mesh has direct contact with the abdominal viscera in the IPOM method, potentially causing adhesions and further complications. The development of pre-peritoneal mesh implants, as used in the ventral-TAPP procedure, stems from the desire to avoid the mesh from having contact with the abdominal organs. In open hernia surgery, procedures in which the inserted meshes have no contact with the visceral organs are recommended [24]. Of note, the lap. IPOM method uses coated meshes, which, as described, are much more expensive, but do not fully protect against adhesions [7]. Recent developments of the lap. IPOM method mention advantages of a primary defect closure [25]. The operations in our study were performed according to the respective current recommendations of the IEHS, which made a corresponding recommendation only in the more recent editions after 2019 [12, 13]. Therefore, primary defect closure was implemented in the latest lap. IPOM and for all ventral-TAPP procedures, since ventral-TAPP was introduced later into our institution.

Ruíz et al. [26] stated that the ventral-TAPP should become the gold standard for incisional hernia in the future. Their study with 59 patients showed few complications. Of the seven patients with complications, there was one case of recurrence, one case of chronic pain and five cases with complications according to the Clavien–Dindo classification [26]. Furthermore, they described extra-peritoneal hernia repair as a cost-effective method, which can be confirmed by our study.

In contrast to Van Hoef et al. [3] we can claim that the alternatives to the lap. IPOM, method (e.g. ventral-TAPP) are by no means more expensive. As our study shows, the material costs of the pre-peritoneal method are much lower (*p* = 0.001). Another point regarding cost-effectiveness is that the length of stay of patients in the ventral-TAPP cohort was significantly lower. We assume that this result is due to the reduced pain after ventral-TAPP, the lower need for painkillers and the resulting faster mobilization of these patients. This represents an indirect cost reduction, as it saves on material and personnel costs.

Of note, this type of surgery (lap. IPOM and ventral-TAPP) is also offered as an outpatient service; the German healthcare system is designed for inpatient procedures with a defined minimal stay of 2 days in this regard, so that these remarks are exclusively representative for Germany. Our experience of the postoperative course after ventral-TAPP was, that many patients would be suitable for a day-surgery procedure.

One of the criticisms of the ventral-TAPP method is that it takes longer to operate than the lap. IPOM method [15]. According to our data, we cannot confirm this. The surgery times in our study showed no statistical difference (65.19 min mean in lap. IPOM vs. 58.65 in ventral-TAPP). However, we can agree that the TAPP technique is technically more demanding and laparoscopic pre-peritoneal mesh placement is a method for more experienced laparoscopists [11, 27, 28]. Since ventral-TAPP repairs were performed by experienced laparoscopists in our hospital, no conversion to lap. IPOM procedure was necessary. We recognize, that larger defects of peritoneum during ventral-TAPP tissue preparation could make the placement of a standard mesh impossible. Therefore, a surgeon who treats ventral hernia using ventral-TAPP must have undergone in general more laparoscopy training compared to lap. IPOM procedure.

In the literature, direct relationship between mesh fixation by tacks during IPOM procedure and postoperative pain has been demonstrated [29, 30]. Surprisingly, Prasad et al. [14, 15] did not find any significance in their studies with regard to postoperative abdominal pain. On the other hand, a study by Ngo et al. [27] with 98 patients after laparoscopic pre-peritoneal mesh placement showed similar results to our study regarding the VAS score. In this analysis, the need for comparative studies was pointed out, which we have done with the present work. We would like to emphasize at this point that the postoperative pain was significantly lower in our ventral-TAPP group compared to lap. IPOM patients. In our view, this is likely due to the fact, that no tissue tackers were used and the mesh was gently positioned in the pre-peritoneal space. Furthermore, our evaluation of the opiates administered postoperatively also showed significance (*p* = 0.001). More precisely, the number of patients who needed postoperative opiates for pain management was four times higher in the lap. IPOM group than in the ventral-TAPP group (*n* = 14 vs *n* = 3, *p* = 0.001). A likely reason for this, is the use of transfascial sutures in the lap. IPOM group, as these non-resorbable sutures are associated with more pain in the first 6 weeks after the operation as compared to mesh fixation with metal tacks [31].

In the robotic study by Gokcal et al. [14], 38.5% of the hernias in the robotic ventral-TAPP group were treated using lap. IPOM mesh (Symbotex™, Medtronic™). The question here is whether this was due to the lack of haptic feedback and a consecutive lesion of the peritoneum. In our study, the peritoneum was always closed with sutures and a simple, uncoated polypropylene mesh was placed. This causality certainly shows again one of the disadvantages of pre-peritoneal mesh placement: as laparoscopic preparation of the peritoneum requires training of the surgeon in this field.

The study by Kumar et al. [32] also shows results favoring pre-peritoneal mesh placement. The hernia size in this study is comparable to ours, but the operation times of the e-TEP method are almost two times longer than the ventral-TAPP of our study (107.52 ± 23.44 min versus 57.61 ± 18.36 min) [32]. At this regard, ventral-TAPP offers the surgeon a better view over the surgical site allowing a faster tissue preparation and defect closure. Besides that, in our study we took advantage of a recently developed simple extra-corporal sliding knot described in patent WO2016/005118 A1 to suture the hernia defect. This technique by-passes with high security the need for time-consuming extra or intra-corporal knot tying within small spaces associated with laparoscopic surgery. Furthermore, the e-TEP method for this type of hernia (small to medium-sized ventral hernias) showed in study by Kumar two recurrences in 46 cases [32]. Here, the ventral-TAPP method seems to be superior, as neither our cohort nor the two by Gokcal et al. and Prasad et al. showed recurrences [14, 15, 32].

Finally, it should be mentioned that in all these studies, small to medium-sized hernias were treated [14, 15, 32]. In our opinion, the treatment of large hernias by means of minimally invasive pre-peritoneal mesh placement in ventral-TAPP technique will be difficult.

A current consideration in the treatment of ventral hernias is the more precise differentiation between primary hernias and incisional hernias. A systematic review and meta-analysis by Stabilini et al. supports the hypothesis that primary hernias and incisional hernias are different conditions with the latter being more challenging to treat [33]. According to this analysis, the difficulty of surgical treatment of incisional hernias often arises from the size and diverse nature of these hernias. One finding that emerges from this extensive and detailed work is that primary hernias appear to be smaller and are more common in younger, healthier patients than incisional hernias. In our study, we included only smaller hernias within propensity matched groups considering several parameters as age, hernia diameter, ASA score and BMI, so we assume that differentiation between primary and incisional hernia are less impactful on our analysis results. Nevertheless, we agree that in larger hernias a distinction should be made between primary and incisional hernias and that this aspect should definitely be addressed in prospective studies.

Limitations of our study are the non-randomized protocol of the study design. Furthermore, the number of patients is small. To overcome these issues our study was performed using propensity-score matched cohorts. To have more information on hernia recurrence a longer observation time after surgery is preferable. At this point, it should also be mentioned that the initially larger number of patients in the lap. IPOM group was the result of repairing even larger hernias with this technique over a longer period of time. Preperitoneal mesh placement is limited by the distribution of peritoneal fat at the lower abdomen and the midline, ventral-TAPP repair is not suitable for larger hernia reconstruction. Therefore, the mesh in the ventral-TAPP method was customized to pre-peritoneal space and placed without fixation as in the TAPP technique for inguinal repair. On the other hand, the mesh in the lap. IPOM technique was placed in the original size as defined by the manufacturer´s intended use and fixated with transfascial sutures and tacks.

We excluded from our analysis hernias with a defect size larger than 5 cm. This might represent a form of selection bias in a comparison regarding all hernia sizes at first sight, but anatomic distribution of pre-peritoneal fat does not allow ventral-TAPP repair of larger hernias. Our aim was to create groups as homogeneous as possible under retrospective conditions by focusing on small and mid-sized hernias and using propensity-score matching in our comparison.

The lap. IPOM method is established worldwide [3] and is also used as standard in our clinic by the majority of our staff. The ventral-TAPP method, on the other hand, is an innovative and not yet established method [14]. In our clinic, a small workgroup has been specializing recently in the ventral-TAPP method for the treatment of ventral hernias. Therefore, the ventral-TAPP group represents a more recent group of expertise. Besides the advanced surgical skills during the different timeframes of our study, there were no changes in pain management strategies or other patient care that have influenced results regarding postoperative pain level or length of hospital stay.

The fact that the ventral-TAPP cohort is the most current group is also reflected in the follow-up time, which at 14.70 months in mean is certainly shorter compared to lap. IPOM group, but provides 1 year observation results of a promising technique. During this follow-up period, as our results and the existing literature shows, no recurrence of hernias was observed [14, 15, 32]. We are awaiting prospective randomized studies with longer observation time to show more solid results.

## Conclusion

We were able to confirm the hypotheses of the previous case-series and comparative studies regarding cost-effectiveness and feasibility of the ventral-TAPP procedure. Additionally, we gain some new insights into postoperative outcomes. Especially, our analysis reveals that ventral-TAPP procedure could be an alternative technique to lap. IPOM repair, reducing the risk of complications related to intra-peritoneal position of mesh and fixating devices. In addition, our study showed that postoperative pain level, opiate intake, hospital stay and material costs of ventral-TAPP cohort are significantly lower compared to lap. IPOM treated patients. Our results regarding postoperative pain represent a new finding compared to previous studies in the same topic. Nevertheless, the long-term results of the two methods revealed no statistically relevant differences regarding outcome. To further validate these results, multi-center prospective studies regarding ventral-TAPP repair are necessary.

## Supplementary Information

Below is the link to the electronic supplementary material.

Supplementary file1 (MP4 366247 KB)
